# Impact of Si on C, N, and P stoichiometric homeostasis favors nutrition and stem dry mass accumulation in sugarcane cultivated in tropical soils with different water regimes

**DOI:** 10.3389/fpls.2022.949909

**Published:** 2022-07-29

**Authors:** Milton Garcia Costa, Marcilene Machado dos Santos Sarah, Renato de Mello Prado, Luiz Fabiano Palaretti, Marisa de Cássia Piccolo, Jonas Pereira de Souza Júnior

**Affiliations:** ^1^Laboratory of Plant Nutrition, Department of Agricultural Sciences, São Paulo State University, Jaboticabal, São Paulo, Brazil; ^2^Laboratory of Irrigation, Department of Rural Engineering, São Paulo State University, Jaboticabal, São Paulo, Brazil; ^3^Laboratory of Nutrient Cycling, Center of Nuclear Energy in Agriculture, University of São Paulo, Piracicaba, São Paulo, Brazil

**Keywords:** beneficial element, water deficit, abiotic stress, fertigation with si, *Saccharum officinarum* L

## Abstract

Studies with silicon (Si) in sugarcane indicate a greater response in productivity in plants under stress, and the underlying mechanisms of Si in the crop are poorly reported. In this context, the benefits of Si in the crop’s stem production are expected to occur at the C:N:P stoichiometry level in plant tissues, benefiting plants with and without stress. However, the extension of this response may vary in different soils. Thus, this research aimed to evaluate if fertigation with Si modifies the C:N:P stoichiometry and if it can increase sugarcane’s nutritional efficiency and vegetative and productive parameters. Therefore, three experiments were installed using pre-sprouted seedlings to cultivate sugarcane in tropical soils belonging to the Quartzarenic Neosol, Eutrophic Red Latosol, and Dystrophic Red Latosol classes. The treatments comprised a 2 × 2 factorial scheme in each soil. The first factor was composed without water restriction (water retention = 70%; AWD) and with water restriction (water retention = 35%; PWD). The second factor presented Si concentrations (0 mM and 1.8 mM) arranged in randomized blocks with five replications. Fertigation with Si increases the Si and P concentration, the C and N efficiency, the C:N ratio, and the dry mass production. However, it decreases the C and N concentration and the C:P, C:Si, and N:P ratios in sugarcane leaves and stems regardless of the water regime adopted in the three tropical soils. Cluster and principal components analysis indicated that the intensity of the beneficial effects of Si fertigation on sugarcane plants varies depending on the cultivation soil and water conditions. We found that Si can be used in sugarcane with and without water stress. It changes the C:N:P homeostasis enough to improve the nutritional efficiency of C, P, N, and, consequently, the dry mass accumulation on the stems, with variation in the different cultivated soils.

## Introduction

In tropical soils, the available silicon (Si) concentration in the soil solution is limited mainly by the high degree of weathering, low pH, and high desilication rate resulting from intense leaching (Keeping, 2017), even in high Si concentration on the ground. Tropical soils are predominantly Latosolss, Ultisols, and Entisols, with a predominance of kaolinite in the mineralogical composition (Poppiel et al., 2018) and consequently low available Si content (≤20 mg kg^–1^ of Si) (0.01 M CaCl_2_ extractor) (Haynes, 2014). On the other hand, the levels of available Si can decrease until the polymerization process, forming dimers and short linear oligomers (polysilicic acid) and evolving to the formation of a silica gel (SiO_2_ n.H_2_O), which is unavailable for root absorption (Schaller et al., 2021). Silicon mobility is aggravated in sandy textured soils, such as Quartzarenic Neosol, so the chemical and physical attributes influence the Si dynamics in the soil and its availability to plants.

Evidence indicates greater Si use from the soil solution by Si-accumulating plants, such as sugarcane (*Saccharum officinarum* L.) (Teixeira et al., 2021, 2022), as they have efficient carriers for Si absorption (LSi1, LSi2, and LS6) (Yamaji and Jian Feng, 2009). However, studies on this species and most crops focus on plants grown under abiotic (Verma et al., 2020b; Sousa Junior et al., 2021; Teixeira et al., 2021) and biotic stress conditions (Tomaz et al., 2021). There are indications that the beneficial element modifies the C:N:P stoichiometry, guaranteeing productivity gains, which was observed in forage plants cultivated in Quartzarenic Neosol (Rocha et al., 2021, 2022) and young sugarcane plants cultivated for only 30 days (Teixeira et al., 2020) and 80 days (Oliveira Filho et al., 2021). However, it is unknown whether these effects could reflect in a later growth stage of the crop’s stems. Although this line of investigation is starting in sugarcane, it can be very promising since there may be potential for Si to promote changes in the elemental stoichiometry of C, N, and P, improving the plant’s nutritional processes. It is possible to consider that the Si in plant tissues predominates in the cell wall and has a lower assimilation cost than C (Schaller et al., 2012), which could impact the homeostasis of plant structural elements and consequently the nutritional efficiency of the plant and the production of stems. However, it may also depend on the soil’s Si balance, which needs to be better understood before being proven.

In sugarcane, water deficit is a limiting factor for growth and development. It leads to a productivity drop, which has become more recurrent in recent years (Teixeira et al., 2021; Verma et al., 2021). However, Si use can mitigate the deleterious effects of this water deficit by acting on biochemical and physiological processes already elucidated in the literature (Marchiori et al., 2017; Yan et al., 2018; Jain et al., 2019). On the other hand, most research results were obtained using Si in the form of calcium silicate, which is characteristically insoluble in water (0.01% at 20 *^o^*C) (Camargo and Keeping, 2021). Therefore, it implies high doses in Entisol and Spodosol soils from the United States (McCray and Ji, 2018), Rhodic Hapludox and Quartzarenic Neosol soils from Brazil (Camargo et al., 2019, 2021a,2021b), and clayey-sandy soils without a defined class from China (Verma et al., 2020a,b).

Currently, researchers aim to reduce the amount of Si in crops using soluble sources via fertigation, favoring ion-root contact, increasing the plant’s Si absorption efficiency (Shukla et al., 2018), and providing greater sugarcane productivity (Dingre and Gorantiwar, 2021; Singh et al., 2021). This trend is based on the greater solubility of the source used, which reduces the risk of Si polymerization in the soil since the element concentrations in the solution applied to the soil are lower than the limit (3 mM of Si) in which it starts the polymerization process (Schaller et al., 2012).

In this context, using soluble sources of Si applied via irrigation systems is a promising technique, and fertigation can improve the decrease in the polymerization rate and crop yield under water deficit conditions. According to Frazão et al. (2020), it can even improve sugarcane growth without stress in soilless cultivation using Si solution. However, there is a lack of research on these sources. In addition, it is essential to better understand Si effects in alleviating water stress, knowing the underlying mechanisms involved, since most studies restrict gas exchange assessments (Bezerra et al., 2019; Teixeira et al., 2021), and very few studies delve deeper into the nutritional study.

In this scenario, it is pertinent to evaluate two hypotheses: first, that Si applied via fertigation is efficient and, after absorbed, is sufficient to modify the stoichiometric homeostasis of C, N, and P; and whether it is sufficient to increase nutritional efficiency and, consequently, if it would alleviate the losses in the dry mass production of leaves and stems of sugarcane cultivated under water deficit, or enhance the dry mass production of leaves and stems in the irrigated crop without water deficit, in different types of soil.

If these hypotheses are accepted, it will be proven for the first time that the Si effect via fertigation on the C:N:P elemental stoichiometry is relevant. It favors not only the initial growth but also a more advanced stage in sugarcane. In other words, until stem production achieving agronomic importance. This finding should strengthen the Si agricultural use for the sustainable cultivation of sugarcane in tropical soils, in marginal regions with water restriction, and improve production in irrigated areas (without water restriction), where the cultivation of both sugarcane producing regions is growing in the world. Therefore, this research was carried out to evaluate whether Si fertigation can modify the C:N:P stoichiometry and whether this can increase nutritional efficiency and, consequently, the biomass production of leaves and especially stems of sugarcane cultivated with and without water deficit, depending on the soil.

## Material and methods

### Experimental areas

**FIGURE 1:**
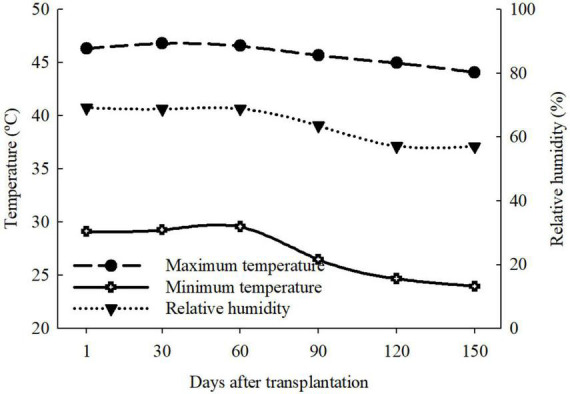
Temperature and relative humidity of the air during the experimental period of cultivation with absence (0.0 mM) and presence of fertigated Si (1.8 mM) and two water conditions (35 and 70%) in three tropical soils (Quartzarenic Neosol; Eutroferric Red Latosol; and Dystrophic Red Latosol).

The study comprised three experiments with the sugarcane variety RB 962860 in a greenhouse at the Universidade Estadual Paulista “Júlio Mesquita Filho” (UNESP), Jaboticabal Campus, Brazil. The three experiments were independent and included three soil types: Quartzarenic Neosol (NQ); Eutrophic Red Latosol (Oxisol) (LVe); and Dystrophic Red Latosol (Oxisol) (LVd). The temperature and relative humidity inside the greenhouse were recorded using a thermo-hygrometer, showing a maximum average temperature of 45.6 ± 1.3°C, minimum of 26.8 ± 2.5°C, and relative humidity of 63.1 ± 6.3% (Figure 1).

### Experimental design

The experimental design used in the three experiments was in randomized blocks, in a 2 × 2 factorial scheme, with two water conditions: without water restriction (water retention = 70%; AWD) and with water restriction (water retention = 35%; PWD); combined with Si absence (0 mM) and presence (1.8 mM), in five repetitions. The experimental plot comprised a pre-sprouted seedling aged 60 days after the bud emergence and inoculated with *Rhyzobium meliloti* in a 20 L polypropylene pot containing 18 L of soil sample during a 150-day cycle. The inoculation of the *Rhizobium meliloti* strain was used to aid in the biological fixation of N. After transplanting, the seedlings were pruned at 0.3 ± 0.02 m from the soil surface, to avoid water loss.

### Installations of experimental plots

The three experiments carried out the collection of soils from the surface layer of a native vegetation area. Then, the soil samples were dried in the open air and sieved with sieves (2 mm). Next, chemical analysis was performed for soil fertility using the method described by Raij et al. (2001). After that, an analysis of the Si concentration was conducted following the method described by Korndorfer et al. (2004). Finally, a granulometric analysis was carried out according to the method indicated by Donagema et al. (2011) (Table 1). The used soils presented sandy, clayey, and sandy loamy textures in Quartzarenic Neosol, Eutrophic Red Latosol, and Dystrophic Red Latosol, respectively.

**TABLE 1:** Chemical characteristics of Quartzarenic Neosol (NQ), Eutroferric Red Latosol (LVe), and Dystrophic Red Latosol (LVd).

Soil	pH	OM	P	S	K	Ca	Mg	Al	H + Al	CTC	V	m	Si	Clay	Silt	Sand
	CaCl_2_	g dm^–3^	mg dm^–3^		mmol_*c*_ dm^–3^						%		mg kg^–1^	g kg^–1^		
NQ	4.3	9	2	6	0.3	3	1	0	16	20.0	21	0	1	50	10	940
Lve	6.2	8	8	8	1.0	16	5	0	17	38.6	57	0	5	550	240	210
LVd	5.2	9	20	7	1.2	14	6	0	22	44.2	49	0	3	300	40	660

pH: CaCl_2_ by potentiometry; H + Al: SMP buffer by potentiometry; OM: Organic matter, by spectrophotometry; P: in resin by spectrophotometry; S: by turbidimetry; K, Ca, and Mg: atomic absorption spectrometry; Si: 0.01 mM calcium chloride, CTC: Cation exchange capacity; V: base saturation; m: Aluminum Saturation.

Soil acidity was corrected 45 days before transplanting, correcting base saturation to 60%. Nutrient supply was performed via fertigation from phosphate fertilization 30 days after liming, nitrogen, potassium, and nitrogen fertilization of micronutrients in transplanting, and the topdressing at 76 days after transplanting N and K. For phosphate fertilization, a dose of 90, 70, and 50 P_2_O_5_ mg dm^–3^ was used for Quartzarenic Neosol, Eutrophic Red Latosol, and Dystrophic Red Latosol, respectively. The dose used for nitrogen fertilization was 15 mg N dm^–3^ and, for K fertilization, 100 mg K_2_O dm^–3^. As for the micronutrients, 2 mg B dm^–3^ and 5 mg Zn dm^–3^ were used.

### Water retention in the soil

The soil water retention capacity was determined for each soil by filling the pots (lysimeters) (20 L) with soil (18 L), with three replications and, later, placing them in a water tank filled with water (2/3 of the height of the pots) for a period of 24 h. Then, the pots were covered with plastic film, drained freely, and their mass was measured at 0, 24, 36, 48, 60, and 72 h after saturation. After draining the excess water, the water replacement capacity was determined through the difference between the wet and dry soil masses. Finally, the treatments’ gravimetric and volumetric moisture, soil density, and gross irrigation depths were calculated (Bernardo et al., 2019).

In each experiment, two lysimeters were installed, one for each level of water retention in the soil (35 and 70%). The daily mass variation of the lysimeters was measured using load cells (model GL 50; Alfa Instruments Electronics S.A.) with a 0.57 mm precision. Data were stored in a data logger (CR10X Campbell Sci., Logan – United States) by connecting a differential channel multiplexer data acquisition system (AM 416 Relay Multiplexer, Campbell Sci., Logan, UT, United States). Data logger data were extracted using the PC200W software interface. Every fifteen days, the measurement of the masses of the lysimeters was performed using a digital scale to adjust possible variations.

The maintenance of soil moisture retention levels lost by evapotranspiration was performed manually every two days for 16–18 h. At 30 days after transplanting, the water retention level in the soil was maintained at 70% in all treatments. Then, two retention levels (35 and 70%) were installed. The installation of the 35% water retention was gradual, first reducing to 50% and, after seven days, to 35% of the soil’s water retention capacity.

### Silicon fertigation

Silicon fertigation was performed every two days using a source of sodium-potassium silicate stabilized with sorbitol at a concentration of 1.8 mM (113.4 g Si L^–1^, 18.9 g K_2_O L^–1^, 100 mL sorbitol L^–1^, and pH 11.8). The pH value of the silicate solution was adjusted to 6.0 ± 0.5 using HCl 1 mM. Finally, potassium balance was performed using KCl fertigation (8.43 mg K L^–1^) in treatments without Si fertigation.

### Analysis

#### Biomass partition

At the end of the cultivation cycle, the cut was carried out at 0.1 m from the soil surface of the sugarcane’s aerial part. After cutting the plants, the samples were separated into leaves and stems and washed with deionized water, detergent solution (0.1% v/v), HCl solution (0.3% v/v), and deionized water, respectively. Then, the samples were dried in an oven with forced air circulation (65 ± 5°C) until reaching constant mass, and, finally, the leaf dry mass (LDM) and stem dry mass (SDM) were determined.

#### C, N, P, and Si concentrations in leaves and stems

The determinations of C and N concentrations were carried out from the dry combustion (1,000°C) of the LDM and SDM using the elemental analyzer (LECO truspec CHNS), calibrated with the LECO 502-278 standard (C = 45.0%). The P concentration was determined from the nitric-perchloric digestion and the spectrophotometer reading (Carmo et al., 2000). The Si concentration was determined from the alkaline digestion (H_2_O_2_ and NaOH) and the reading of the colorimetric reaction with ammonium molybdate using the spectrophotometer (Korndorfer et al., 2004).

#### Stoichiometric ratios and content of C, N, P, and Si

Carbon, Nitrogen, Phosphorus, and Silicon concentrations from leaves and stems were used to establish the C:N, C:P, N:P, and C:Si stoichiometric ratios. The C, N, P, and Si content were estimated for the leaves and stems from the product of the nutrient concentration and the dry mass of the studied organ.

#### C, N, and P use efficiency

The C, N, and P use efficiencies in leaves and stems were estimated from the quotient of the square of dry matter and nutrient content (g of accumulated nutrient) (Siddiqi and Glass, 2008).


(1) $$C\,u\,s\,e\,e\,f\,f\,i\,c\,i\,e\,n\,c\,y\,\left(g^{2}\,g^{-1}\right)=\frac{D\,r\,y\,m\,a\,t\,t\,e\,r^{2}}{C\,c\,o\,n\,t\,e\,n\,t}$$

(2) $$N\,u\,s\,e\,e\,f\,f\,i\,c\,i\,e\,n\,c\,y\,\left(g^{2}\,g^{-1}\right)=\frac{D\,r\,y\,m\,a\,t\,t\,e\,r^{2}}{N\,c\,o\,n\,t\,e\,n\,t}$$

(3) $$P\,u\,s\,e\,e\,f\,f\,i\,c\,i\,e\,n\,c\,y\,\left(g^{2}\,g^{-1}\right)=\frac{D\,r\,y\,m\,a\,t\,t\,e\,r^{2}}{P\,c\,o\,n\,t\,e\,n\,t}$$

### Statistical analysis

Data were submitted to preposition tests (Shapiro–Wilk normality and Levene homogeneity) (Royston, 1995; Gastwirth et al., 2009) and, later, analysis of variance (*p* < 0.05) and, when significant, the comparison test of Tukey mean (*p* < 0.05). In addition, a hierarchical cluster analysis was performed using the Euclidean distance as the similarity coefficient and the single linkage method as the group connection algorithm. Furthermore, principal components analysis (PCA) was performed using the correlation matrix. Finally, statistical analyzes were performed using the Python programming language (version 3.9.7; Python Software Foundation).

## Results

### C, N, P, and Si concentrations

The water deficit did not change the Si concentration in the leaves in the three tropical soils in the absence of fertigation with Si. However, there was a decrease in the stems of sugarcane plants cultivated in Quartzarenic Neosol and Eutrophic Red Latosol (Table 2). Regarding its absence, Si fertigation was efficient in increasing the Si concentration in leaves and stems of plants in treatments with water deficit (PWD) and without water deficit (AWD) in the three tropical soils (Table 2).

**TABLE 2:** C, N, P, and Si concentrations in the leaves and stems of sugarcane cultivated with absence (0.0 mM) and presence of fertigated Si (1.8 mM) and two water conditions (without—AWD and with water deficit—PWD) in three tropical soils (Quartzarenic Neosol; Eutroferric Red Latosol; and Dystrophic Red Latosol).

Water condition	Si	Quartzarenic Neosol		Eutrophic Red Latosol		Dystrophic Red Latosol	
		Leaf	Stem	Leaf	Stem	Leaf	Stem
**C (g kg^–1^)**							
PWD	without	444.55 Aa	410.38 Aa	433.78 Aa	408.62 Aa	437.75 Aa	402.38 Ba
	with	421.12 Ab	407.36 Aa	430.08 Aa	402.58 Ba	426.18 Ab	396.84 Ab
AWD	without	442.40 Aa	408.01 Aa	429.58 Ba	411.20 Aa	434.36 Aa	406.04 Aa
	with	418.12 Ab	407.35 Aa	422.00 Bb	410.68 Aa	421.84 Bb	399.20 Ab
MSD (5%)		7.49	5.57	4.12	6.49	3.95	3.01
**N (g kg^–1^)**							
PWD	without	5.62 Aa	3.38 Aa	5.06 Ba	7.03 Aa	4.08 Aa	5.58 Aa
	with	5.18 Ab	2.13 Bb	4.56 Bb	4.75 Ab	3.34 Ab	4.93 Aa
WD	without	5.86 Aa	2.80 Ba	6.12 Aa	3.62 Ba	3.00 Ba	5.38 Aa
	with	5.12 Ab	2.58 Aa	5.34 Ab	2.78 Bb	2.42 Bb	3.92 Bb
MSD (5%)		0.31	0.32	0.37	0.47	0.45	0.66
**P (g kg^–1^)**							
PWD	without	0.42 Bb	0.48 Bb	0.20 Ba	0.14 Aa	0.59 Ba	0.74 Aa
	with	0.51 Ba	0.68 Ba	0.21 Ba	0.16 Aa	0.61 Ba	0.77 Ba
AWD	without	0.65 Aa	0.71Aa	0.33 Aa	0.15 Aa	0.66 Ab	0.64 Bb
	with	0.66 Aa	0.75 Aa	0.34 Aa	0.15 Aa	0.76 Aa	0.93 Aa
MSD (5%)		0.022	0.054	0.024	0.022	0.070	0.058
**Si (g kg^–1^)**							
PWD	without	2.48 Ab	0.44 Bb	1.26 Ab	0.57 Bb	3.29 Ab	0.52 Ab
	with	7.65 Aa	2.32 Ba	6.90 Ba	4.21 Aa	6.79 Ba	1.63 Ba
AWD	without	2.43 Ab	1.42 Ab	3.14 Ab	1.08 Ab	3.16 Ab	0.42 Ab
	with	11.78 Aa	3.26 Aa	12.24 Aa	4.21 Aa	11.22 Aa	2.88 Aa
MSD (5%)		4.68	0.49	2.50	0.50	1.93	0.28

Different uppercase letters indicate differences in water conditions and different lowercase letters indicate differences in Si fertigation by Tukey test (*p* < 0.05); MSD, minimum significant difference.

The C concentration in the leaves increased with the water deficit in sugarcane plants without fertigation with Si in Eutrophic Red Latosol. At the same time, there was a decrease in the C concentration in the stems in Dystrophic Red Latosol under PWD with the absence of fertigation with Si (Table 2). Regarding its absence in sugarcane cultivation, Si fertigation decreased the foliar concentration of C in PWD in Quartzarenic Neosol and Dystrophic Red Latosol. While in Eutrophic Red Latosol, there was no difference (Table 2). On the other hand, under AWD conditions, Si fertigation decreased the foliar concentration of C in plants cultivated in the three tropical soils (Table 2). In turn, the C concentration in the stems also decreased with Si fertigation only in Dystrophic Red Latosol and in both water retention capacities (PWD and AWD) (Table 2).

The water deficit decreased the N concentration in the plant’s leaves in the absence of fertigation with Si in Eutrophic Red Latosol. While in Dystrophic Red Latosol, there was an increase in this nutrient’s concentration (Table 2). Regarding the stems, there was an increase in the N concentration with the absence of fertigation with Si in sugarcane plants cultivated in Quartzarenic Neosol and Eutrophic Red Latosol (Table 2). However, the N concentration in the leaves decreased with the Si fertigation regarding its absence in the plants cultivated in two water conditions and the three tropical soils (Table 2). In the stem, it was verified that the Si fertigation regarding its absence in PWD caused a decrease in the N concentration in the stems of plants in the cultures carried out in Eutrophic Red Latosol and Dystrophic Red Latosol. However, there were no differences in Quartzarenic Neosol. In addition, plants grown in AWD and fertigated with Si decreased stem N concentration only in Eutrophic Red Latosol (Table 2).

Phosphorus concentration in leaves and stems decreased with the water deficit in sugarcane plants without Si fertigation in Quartzarenic Neosol and Eutrophic Red Latosol. However, there was an increase in the P concentration in Dystrophic Red Latosol (Table 2). The increase in the P concentration in leaves and stems was observed in PWD fertigated with Si regarding its absence in sugarcane cultivation only in Quartzarenic Neosol (Table 2). Meanwhile, there was an increase in the P concentration in the leaves and stems of plants under AWD conditions promoted by the fertigation of Si, regarding its absence, but only in plants cultivated in Dystrophic Red Latosol (Table 2).

### Stoichiometric ratios of C:N, C:P, C:Si, and N:P

**FIGURE 2:**
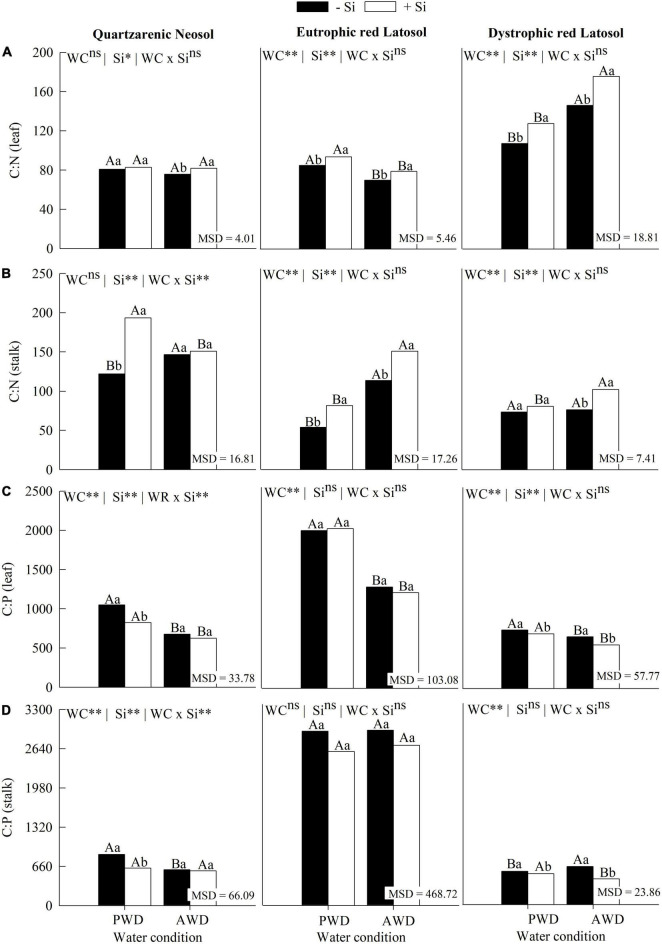
Stoichiometric ratios of C:N **(A,B)** and C:P **(C,D)** in leaves and stems of sugarcane cultivated with absence (0.0 mM) and presence of fertigated Si (1.8 mM) and two water conditions (without—AWD and with water deficit—PWD) in three tropical soils (Quartzarenic Neosol; Eutroferric Red Latosol; and Dystrophic Red Latosol). WC, water condition; MSD, minimum significant difference; ** and *: significant at 1 and 5% probability, respectively; NS = not significant at 5% probability. Different uppercase letters indicate differences in water conditions and different lowercase letters indicate differences in Si fertigation by Tukey’s test (*p* < 0.05).

The C:N ratio in leaves increased with the water deficit in sugarcane plants without Si fertigation in Eutrophic Red Latosol. However, there was a decrease in this ratio in plants cultivated in Dystrophic Red Latosol (Figure 2A). Additionally, there was a decrease in the C:N ratio in the leaves of plants grown in the absence of fertigation with Si in Quartzarenic Neosol and Eutrophic Red Latosol (Figure 2B). On the other hand, Si fertigation increased the C:N stoichiometric ratio in the plants’ leaves in the two water conditions and the three tropical soils, except for the PWD plant grown in Quartzarenic Neosol (Figure 2A). This stoichiometric alteration was also observed in stems, with similar behavior in the plant’s leaves, except for PWD in Dystrophic Red Latosol cultivation and AWD in Quartzarenic Neosol cultivation (Figure 2B).

The water deficit increased the C:P ratio in the leaves of plants cultivated in the three tropical soils without Si fertigation (Figure 2C). There was also an increase and decrease in this ratio in this organ on the crops cultivated in Quartzarenic Neosol and Dystrophic Red Latosol, respectively (Figure 2D). Furthermore, Si application decreased the C:P stoichiometric ratio in leaves and stems of sugarcane cultivated under PWD in Quartzarenic Neosol and under PWD and AWD in Dystrophic Red Latosol (Figures 2C,D).

**FIGURE 3:**
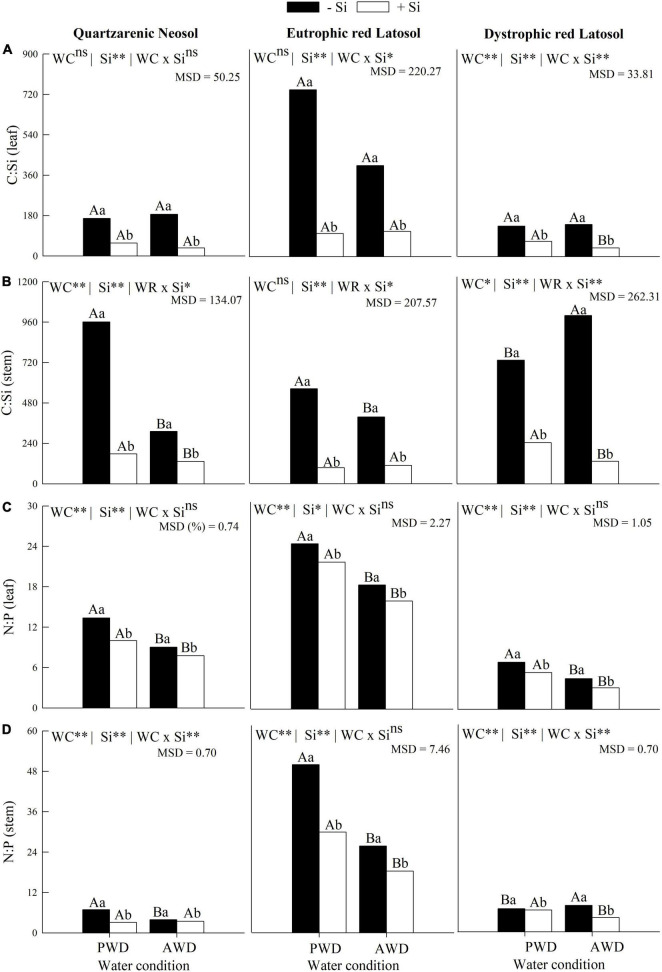
Stoichiometric ratios of C:Si **(A,B)** and N:P **(C,D)** in leaves and stems of sugarcane cultivated with absence (0.0 mM) and presence of fertigated Si (1.8 mM) and two water conditions (without—AWD and with water deficit—PWD) in three tropical soils (Quartzarenic Neosol; Eutroferric Red Latosol; and Dystrophic Red Latosol). WC, water condition; MSD, minimum significant difference; ** and *: significant at 1 and 5% probability, respectively; NS, not significant at 5% probability. Different uppercase letters indicate differences in water conditions and different lowercase letters indicate differences in Si fertigation by Tukey’s test (*p* < 0.05).

The water deficit did not change the C:Si ratio in the leaves of sugarcane plants without Si fertigation in the three tropical soils (Figure 3A). However, there was an increase in the C:Si ratio in Quartzarenic Neosol and a decrease in this ratio in Eutrophic Red Latosol and Dystrophic Red Latosol (Figure 3B). Silicon use decreased the C:Si stoichiometric ratio in leaves and stems of sugarcane cultivated under PWD and AWD in the three tropical soils (Figures 3A,B).

There was an increase in the N:P ratio in leaves and stems with the water deficit in plants without Si fertigation in the three tropical soils (Figures 3C,D), except for sugarcane stems cultivated in Dystrophic Red Latosol (Figure 3D). Moreover, there was also a decrease in the leaf stoichiometric ratio of N:P in the plant, with the fertigation of Si in the two water conditions and two soils (Quartzarenic Neosol and Dystrophic Red Latosol) (Figure 3C). This ratio’s stoichiometric ratio in the stem decreased in PWD in plants grown in Quartzarenic Neosol and Eutrophic Red Latosol. It also decreased in the AWD condition of the culture grown in the two Latosols (Figure 3D).

### Si, C, N, and P contents

Water deficit decreased Si content in leaves and stems in sugarcane plants without Si fertigation in the three tropical soils, except for plants cultivated in Dystrophic Red Latosol (Table 3). Conversely, Si fertigation increased Si content in leaves and stems in the two water conditions studied and the three tropical soils (Table 3).

**TABLE 3:** C, N, P, and Si content in leaves and stems of sugarcane cultivated with absence (0.0 mM) and presence of fertigated Si (1.8 mM) and two water conditions (with and without water deficit) in three tropical soils (Quartzarenic Neosol; Eutroferric Red Latosol; and Dystrophic Red Latosol).

Water condition	Si	Quartzarenic Neosol		Eutrophic Red Latosol		Dystrophic Red Latosol	
		Leaf	Stem	Leaf	Stem	Leaf	Stem
**C (g per plant)**							
PWD	without	15437 Ba	6503.9 Bb	7506.4 Bb	4461.5 Bb	11396 Ba	5574.2 Bb
	with	15789 Ba	9563.8 Ba	7819.8 Ba	5911.9 Ba	12100 Ba	6070.0 Ba
AWD	without	16820 Aa	9899.8 Ab	10255 Aa	6769.8 Ab	15030 Ab	8390.1 Aa
	with	16458 Aa	12567 Aa	10729 Aa	7604.8 Aa	16845 Aa	8674.3 Aa
MSD (5%)		440.03	1104.14	250.13	310.58	730.53	372.37
**N (g per plant)**							
PWD	without	193.36 Ba	53.205 Ba	88.825 Ba	74.606 Aa	102.90 Aa	78.100 Ba
	with	192.46 Aa	49.854 Ba	83.721 Ba	67.644 Ab	94.725 Aa	75.562 Aa
APW	without	224.42 Aa	67.663 Ab	146.37 Aa	55.626 Ba	102.95 Aa	114.76 Aa
	with	204.41 Ab	81.817 Aa	138.68 Aa	46.574 Bb	93.494 Aa	85.525 Ab
MSD (5%)		12.36	9.05	8.63	5.29	11.97	10.15
**P (g per plant)**							
PWD	without	14.47 Bb	7.579 Bb	3.65 Ba	1.56 Bb	14.85 Bb	10.15 Ba
	with	18.90 Ba	15.69 Ba	3.85 Ba	2.31 Ba	17.43 Ba	11.71 Ba
AWD	without	24.68 Aa	17.23 Ab	7.87 Aa	2.44 Aa	22.64 Ab	13.08 Ab
	with	26.11 Aa	22.50 Aa	8.70 Aa	2.95 Aa	30.30 Aa	19.40 Aa
MSD (5%)		0.98	2.40	0.89	0.56	2.27	1.77
**Si (g per plant)**							
PWD	without	80.02 Bb	7.02 Bb	47.82 Bb	7.71 Bb	84.20 Ab	7.21 Ab
	with	411.73 Ba	47.77 Ba	167.04 Ba	61.42 Ba	192.61 Ba	24.92 Ba
AWD	without	113.21 Ab	28.86 Ab	117.58 Ab	17.69 Ab	118.15 Ab	7.51 Ab
	with	471.12 Aa	91.42 Aa	474.51 Aa	86.0 Aa	467.88 Aa	65.86 Aa
MSD (5%)		26.60	4.34	25.42	9.44	44.21	4.04

Different uppercase letters indicate differences in water conditions and different lowercase letters indicate differences in Si fertigation by Tukey test (*p* < 0.05); MSD, minimum significant difference.

Carbon content in leaves and stems decreased with the water deficit in the three tropical soils without Si fertigation (Table 3). Additionally, Si supply increased leaf C content in the PWD plant grown in Eutrophic Red Latosol. However, it decreased in the plant grown in Quartzarenic Neosol and Dystrophic Red Latosol (Table 3). In AWD, there was an increase in leaf C content by Si fertigation only in cultivation with Dystrophic Red Latosol, with no difference in this variable in the other soil types (Table 3). The C content in the plant stem increased in PWD fertigated with Si in the three tropical soils. In contrast, in AWD, the increase was observed only in the cultivation of Quartzarenic Neosol and Eutrophic Red Latosol (Table 3).

Water deficit also decreased the N content in leaves and stems in sugarcane plants without Si fertigation in the three tropical soils, except for stems in plants cultivated in Eutrophic Red Latosol and leaves in plants cultivated in Dystrophic Red Latosol (Table 3). On the other hand, Si fertigation did not change leaf N content in the two water retention capacities in the three tropical soils, except for Quartzarenic Neosol, where there was a decrease in N content in PWD (Table 3). Additionally, the N content in the stem decreased in PWD fertigated with Si in Eutrophic Red Latosol, while in AWD, there was an increase in this variable in Quartzarenic Neosol and a decrease in Dystrophic Red Latosol (Table 3).

Phosphorus content in leaves and stems of plants was reduced with the water deficit in sugarcane without Si fertigation (Table 3). Furthermore, leaf P content was increased in the plant with Si fertigation in PWD in Quartzarenic Neosol and Dystrophic Red Latosol, and in AWD in Dystrophic Red Latosol (Table 3). Moreover, the P content in the stem was increased as a function of Si fertigation in the plant cultivated in PWD in Dystrophic Red Latosol and Quartzarenic Neosol, and in AWD in Dystrophic Red Latosol (Table 3).

### C, N, and P use efficiency and biomass partition

**FIGURE 4:**
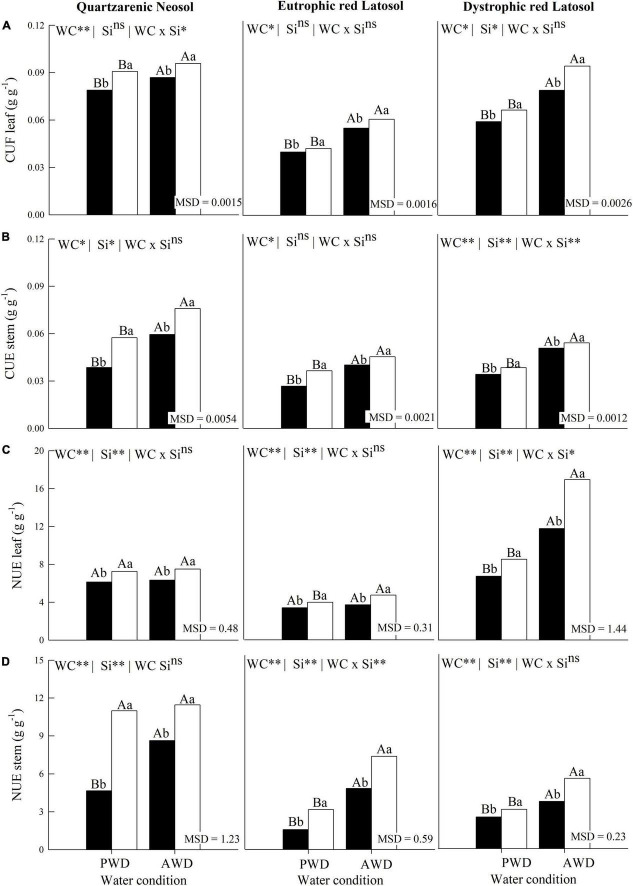
Use efficiencies of Carbon **(A,B)** and Nitrogen **(C,D)** in leaves and stems of sugarcane cultivated with absence (0.0 mM) and presence of fertigated Si (1.8 mM) and two water conditions (without—AWD and with water deficit—PWD) in three tropical soils (Quartzarenic Neosol; Eutroferric Red Latosol; and Dystrophic Red Latosol. CUE, C use efficiency; NUE, N use efficiency; WC, water condition; MSD, minimum significant difference; ** and *: significant at 1 and 5% probability, respectively; NS, not significant at 5% probability. Different uppercase letters indicate differences in water conditions and different lowercase letters indicate differences in Si fertigation by Tukey’s test (*p* < 0.05).

The water deficit reduced the C use efficiency in the leaves and stems of plants grown in the three tropical soils without Si fertigation (Figures 4A,B). Additionally, the C use efficiency in the leaves (Figure 4) and in the stems (Figure 4), and the N use efficiency in the stems (Figure 4), were increased in the plant with Si fertigation in the two water conditions, and the three tropical soils (Figures 4A,B).

The N use efficiency decreased with the water deficit in sugarcane plants without Si fertigation in the three tropical soils, except for leaves cultivated in Quartzarenic Neosol and Eutrophic Red Latosol (Figures 4C,D). Silicon fertigation also increased the efficiency of N foliar use in plants cultivated in the two water conditions and the three evaluated soils, except for Eutrophic Red Latosol cultivated in PWD (Figure 4C).

**FIGURE 5:**
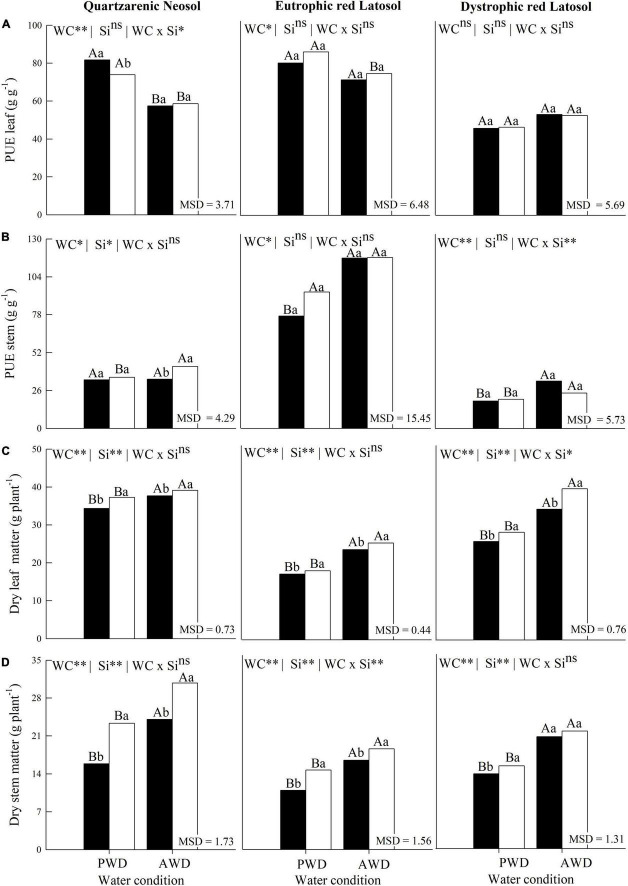
Phosphorus use efficiencies **(A,B)** and dry mass partition **(C,D)** in leaves and stems of sugarcane cultivated with absence (0.0 mM) and presence of fertigated Si (1.8 mM) and two water conditions (without—AWD and with water deficit—PWD) in three tropical soils (Quartzarenic Neosol; Eutroferric Red Latosol; and Dystrophic Red Latosol). PUE, P use efficiency; WC, water condition; MSD, Minimum significant difference; ** and *: significant at 1 and 5% probability, respectively; NS, not significant at 5% probability. Different uppercase letters indicate differences in water conditions and different lowercase letters indicate differences in Si fertigation by Tukey’s test (*p* < 0.05).

Water deficit increased P use efficiency in sugarcane plants without Si fertigation in Quartzarenic Neosol. However, there was decreased P use efficiency in stems in Eutrophic Red Latosol and Dystrophic Red Latosol (Figure 5A). In PWD, there was a decrease in the P use efficiency in leaves in sugarcane fertigated with Si and cultivated in Quartzarenic Neosol. At the same time, there were no differences in this variable in plants cultivated in other soils (Figure 5A). Finally, in the culm, the P use efficiency increased in the plant cultivated in AWD condition in Quartzarenic Neosol. However, this variable did not differ in the other soils (Figure 5B).

The water deficit also decreased dry mass in leaves and stems in the three tropical soils cultivated without Si fertigation (Figures 5C,D). Additionally, leaf dry mass production increased in plants cultivated with Si fertigation in both water conditions and the studied soils (Figure 5C). There was also an increase in dry mass production regarding the plant stem using Si fertigation in both water conditions, except for the AWD plant cultivated in Dystrophic Red Latosol, where there was no effect of Si application for this variable (Figure 5D).

### Hierarchical cluster analysis

**FIGURE 6:**
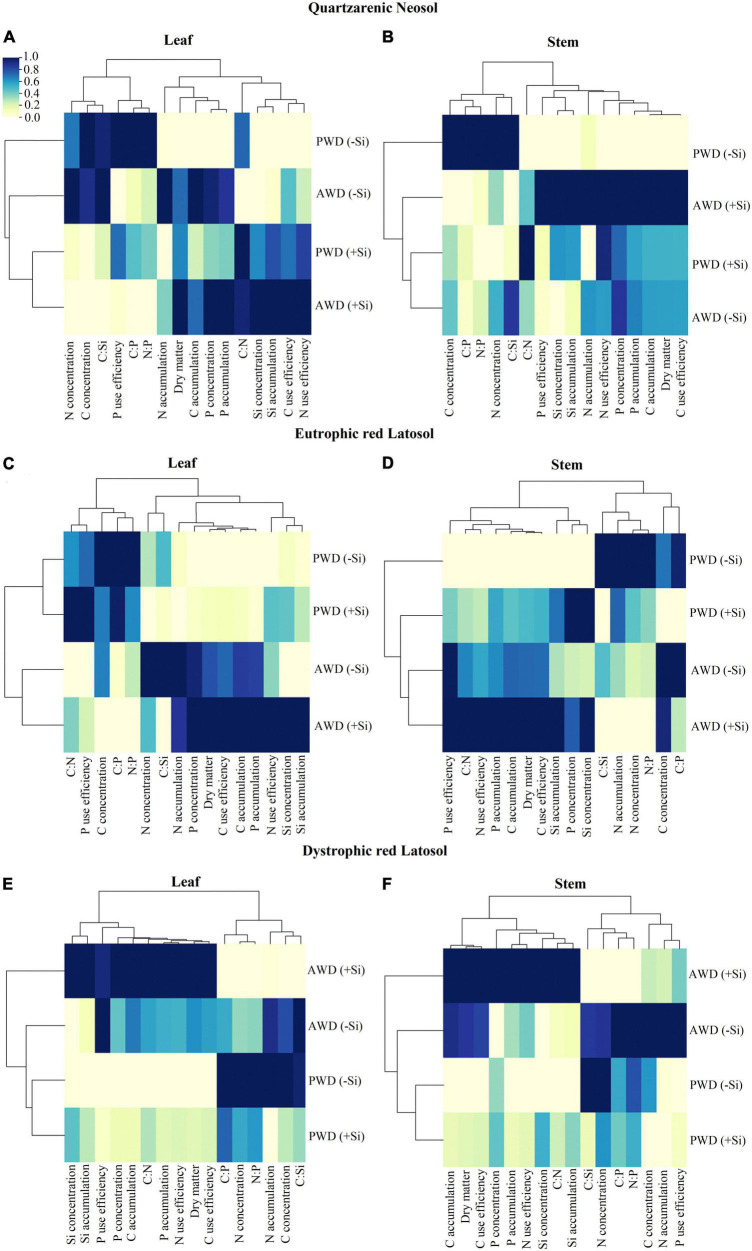
Heat map of hierarchical clustering of variables of concentrations and accumulations of C, N, P, and Si, stoichiometric ratios of C:N:P:Si, use efficiencies of C, N, and P, and dry mass partition in the leaf **(A,C,E)** and in the stem **(B,D,F)** of sugarcane cultivated with absence (0.0 mM) and presence of fertigated Si (1.8 mM) and two water conditions (without—AWD and with water deficit—PWD) in three tropical soils (Quartzarenic Neosol; Eutroferric Red Latosol; and Dystrophic Red Latosol).

The hierarchical cluster analysis for leaves indicated that under water restriction conditions, without Si fertigation, there was greater dissimilarity from the other conditions evaluated in Quartzarenic Neosol. On the other hand, in Eutrophic Red Latosol, two groups were formed, dividing the two water conditions. In Dystrophic Red Latosol, the greatest dissimilarity was observed in the AWD treatment with Si fertigation (Figures 6A,C,E).

There were similar groups in leaves and stems for response variables, however, with a difference in the groups’ composition for tropical soils. For Quartzarenic Neosol, two groups were formed in leaves and stems: the first group comprised the subgroup of N and C concentrations and the C:Si ratio; and the second group comprised the P use efficiency and the C:P and N:P ratios. The second group also comprised two subgroups: the first subgroup comprising the content of C, N, P, the dry mass production, and the P concentration; and the second subgroup comprising the C:N ratio, the efficiency of C and N use, and the Si concentration and content, except for the P use efficiency, which was grouped in the second group in stems (Figures 6A,B).

In Eutrophic Red Latosol, there were two groups for leaves and stems: the first group indicated greater similarity between the C:N, C:P, and N:P ratios, the P use efficiency, and the C concentration; the second group comprised two subgroups, one with the N concentration and the C:Si ratio, and the second with the P and Si concentrations and the C, N, P, and Si contents, due to the C and N use efficiency and the dry mass production. However, there was a change in the C:N stoichiometric ratio and the P use efficiency for the second group in stems (Figures 6C,D). For Dystrophic Red Latosol, the hierarchical grouping analysis of the variables in leaves showed the formation of two groups: the first group indicated greater similarity of the Si concentration and content with the dry mass production, C, N, and P use efficiency, the P concentration, C and P content, and the C:N stoichiometric ratio; the second group comprised the C:P, N:P, and C:Si ratios and the C and N concentrations. However, there was a change in the P use efficiency for the first group in stems (Figures 6E,F).

The hierarchical cluster analysis indicated a greater dry mass production similarity with AWD treatment fertilized with Si in leaves and stems in the three tropical soils. It also found that Si fertigation in PWD showed similar results under AWD conditions in leaves and stems in Quartzarenic Neosol, and stems in Eutrophic Red Latosol (Figure 6). The C and N use efficiency and the P and Si concentration and content are highly related to the AWD treatment with Si fertigation on leaves and stems in the three tropical soils (Figure 6).

The stoichiometric ratios C:Si, C:P, and N:P are inversely proportional to the dry mass production, which showed high similarity with AWD treatment fertigated with Si in leaves and stems in tropical soils (Figure 6). The P use efficiency in the leaves is inversely related to the AWD treatment fertigated with Si in the three tropical soils due to the increase in foliar P concentration (Figure 6). On the other hand, the P use efficiency and concentration in stems showed high similarity with AWD treatment fertigated with Si (Figure 6C).

### Principal component analysis

**FIGURE 7:**
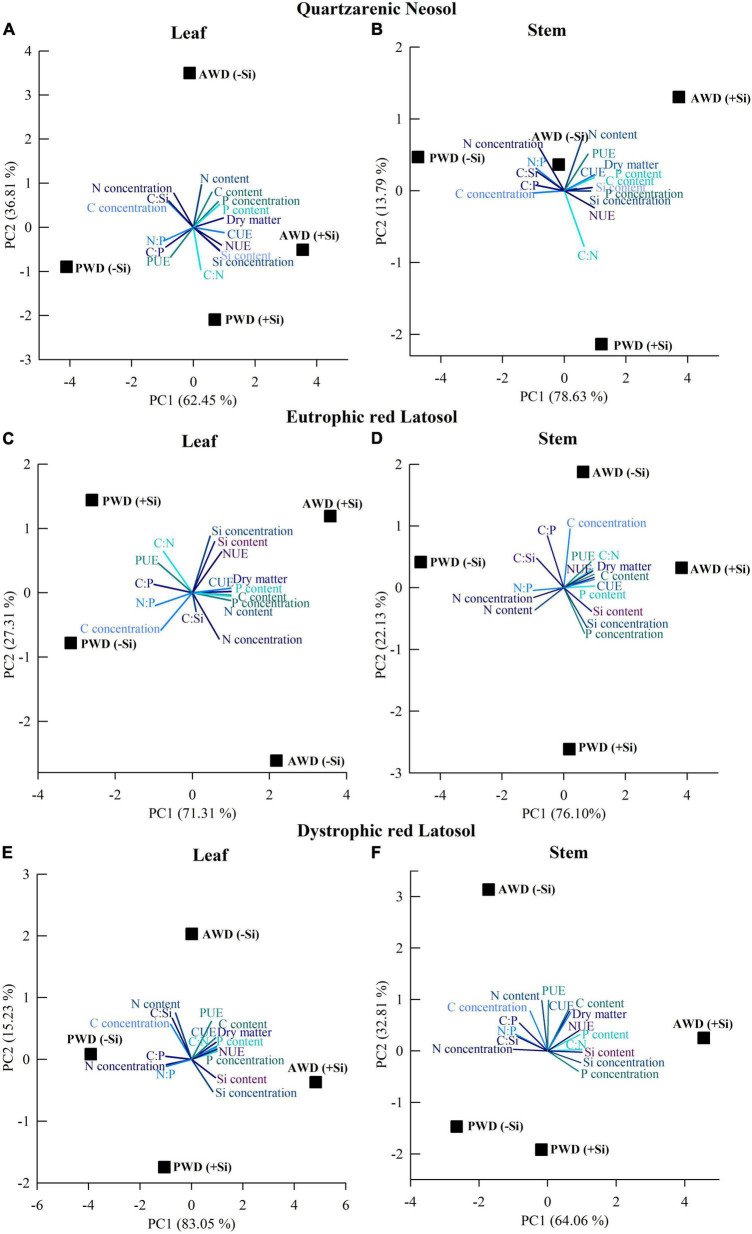
Principal component analysis of the variables of concentrations and accumulations of C, N, P, and Si, stoichiometric ratios of C:N:P:Si, use efficiencies of C, N, and P, and dry mass partition in the leaf **(A,C,E)** and in the stem **(B,D,F)** of sugarcane cultivated with absence (0.0 mM) and presence of fertigated Si (1.8 mM) and two water conditions (without—AWD and with water deficit—PWD) in three tropical soils (Quartzarenic Neosol; Eutroferric Red Latosol; and Dystrophic Red Latosol). CUE, C use efficiency; NUE, N use efficiency; PUE, P efficiency use.

Principal component analysis (PCA) in leaves and stems explained 99.3 and 92.4, 98.6 and 98.2, and 98.1 and 96.9% of the variable responses of sugarcane cultivated in Quartzarenic Neosol, Eutrophic Red Latosol, and Dystrophic Red Latosol, respectively (Figure 6). In the stem, PCA explained 92.4, 98.2, and 96.9% of the response variables of sugarcane cultivated in Quartzarenic Neosol, Eutrophic Red Latosol, and Dystrophic Red Latosol, respectively (Figure 7). Given the importance of stems in crop productivity, we will highlight the PCA analysis in this organ.

In sugarcane cultivation in Quartzarenic Neosol, the C concentration is associated with the PWD treatment without Si fertigation and the C:N ratio with the PWD treatment with Si fertigation (Figure 7B). The N use efficiency, the Si concentration, and the C, P, and Si content were associated with the two water treatments fertigated with Si, and the C and P use efficiency, the N content, and the dry mass production were associated with AWD treatment fertigated with Si (Figure 7B). In addition, PCA indicated that the increase in the C:Si, C:P, and N:P stoichiometric ratios and the N concentration are associated with treatments that were not fertigated with Si in the two water conditions studied (Figure 7B).

For Eutrophic Red Latosol, there was an association of the C:Si and C:P stoichiometric ratios with the treatments not fertigated with Si in the two water conditions studied, while the Si and P concentration and the Si content are associated with the fertigated treatments in the two water conditions (Figure 7D). Furthermore, carbon concentration was associated with PWD treatment without Si fertigation, and the N:P ratio and N concentration and content were associated with PWD treatment without Si fertigation (Figure 7D). Moreover, there was an association between the C, N, and P use efficiency, the C:N ratio, the C and P content, and the dry mass production with the AWD treatment fertigated with Si (Figure 7D).

For the sugarcane stem cultivated in Dystrophic Red Latosol, PCA indicated an association of increased N concentration and C:Si, N:P, and C:P stoichiometric ratios with treatments without Si fertigation. However, N use efficiency, P and Si concentration, P and Si content, and the C:N stoichiometric ratio were associated with treatments fertigated with Si in both water conditions (Figure 7F). Carbon and Nitrogen concentrations and P use efficiency were associated with PWD treatment without Si fertigation, and C use efficiency, C content, and dry mass production were associated with AWD treatment (Figure 7F).

## Discussion

There is a predominance of studies regarding water deficit in sugarcane crops without Si supply. They have reported losses in crop productivity, which is generally explained by damage to the plant’s physiological aspects (Bezerra et al., 2019; Camargo et al., 2019; Teixeira et al., 2021). These water deficit losses in the production of sugarcane stems were also verified in the three soils studied. However, we found that the water deficit caused these losses because there was a change in the C:N, C:P, and N:P stoichiometric ratios, which varied according to the plant’s organ and soil. These effects on these ratios resulted in a decrease in the use efficiencies of C and N in leaves and C and N in stems in Eutrophic Red Latosol and Quartzarenic Neosol. Furthermore, there was a reduction in the C and N use efficiency in leaves and stems in Dystrophic Red Latosol (Figures 2–4), which explains the decrease in the production of sugarcane stems. Therefore, the underlying damage of water deficit is associated with the instability of stoichiometric homeostasis, which causes a decrease in nutritional efficiency and, consequently, in the production of stems. It is not only restricted to losses in gas exchange, as reported by other authors (Marchiori et al., 2017; Bezerra et al., 2019; Camargo et al., 2019; Teixeira et al., 2021). Our results present implications for researchers who study biological damage from water deficit in sugarcane and other species that normally neglect stoichiometric nutrient homeostasis assessments. These facts make it difficult to better understand the nutritional component of crop productivity. Thus, further research is required.

Silicon use is a known alternative to alleviate the water deficit in sugarcane crops, but this may depend on the crop’s ability to absorb this element. This fact favors sugarcane because it is classified as a plant that accumulates Si, having specific transporters in the roots and high efficiency in absorbing this beneficial element (Wang et al., 2021). Additionally, Si is absorbed by LSi influx channels and transported to other tissues and organs by efflux channels, and the cooperative influx and efflux system regulates plant accumulation patterns (Trejo-Téllez et al., 2022). However, soil mineralogy can alter the availability of Si in the soil solution, with greater adsorption of monosilicic acid to the mineral’s hematite, goethite, magnetite, lepidocrocite, akaganeite, feroxyhyte, ferrihydrite and amorphous iron hydroxide, predominant in tropical soils (Dietzel, 2002; Schaller et al., 2021). Under these conditions, the availability of Si in the soil solution is low, reducing plant uptake and, consequently, its benefits (Schaller et al., 2021).

In order to ensure adequate silicate nutrition for the crop, most studies have evaluated its effects on stem production using Si in solid form with sources of very low solubility in water (calcium silicate), implying the use of the element in high doses (Camargo et al., 2017, 2019, 2020; Clemente et al., 2019). However, there were doubts whether fertigation with Si in concentrations diluted with soluble sources. In other words, using relatively low doses could efficiently increase the element’s uptake by the plant. This study answered this question because we showed that fertigation with Si at a concentration of 1.8 mM in both water conditions efficiently increased the element’s content in the plant, that is, its uptake (Tables 2, 3). This finding should change the philosophy of supplying Si in the sugarcane crop for fertigation because using soluble sources through well-diluted Si solutions can reduce the amount of the element applied and directly impact the best cost ratio/benefit of using Si in agriculture.

It is important to highlight that the soil’s water condition also influenced the Si fertigation efficiency. It led to a higher content of the element in the leaves and stems of plants without water deficit. In other words, plants under water restriction presented a loss in Si uptake (Tables 2, 3). The lower soil water availability can decrease the Si content in the root (Shukla et al., 2018). Furthermore, the decrease in the soil’s water content causes an effect on the Si concentration in the soil solution, which can reach a Si concentration above 3 mM, resulting in possible polymerization of part of this element, with a consequent decrease in the availability in the soil for plant absorption (Schaller et al., 2021). However, even under water deficit regarding the control plants, there was the absorption of Si in the plants, a fact also verified in other studies on young sugarcane plants (Teixeira et al., 2021), a necessary condition for the element to benefit the plant.

The Si supply in the soil can also positively affect the nutrients uptake, especially P in sugarcane in the stem production phase. However, these effects are almost unknown when the beneficial element is supplied via fertigation in different soil water regimes. The presence of Si in the soil can compete for binding sites in minerals, decreasing P uptake, consequently increasing this nutrient’s availability for plants (Schaller et al., 2019). Phosphorus is highly adsorbed in tropical soils, resulting in its low availability for plants (Hanyabui et al., 2020). However, Si has increased this nutrient’s mobility in soils.

An interesting Si effect was observed regarding P absorption in the plant. The nutrient content in the leaves and stems promoted by the Si supply increased in plants cultivated in both water regimes, especially in Dystrophic Red Latosol and Quartzarenic Neosol (Table 2). This result evidenced the synergistic effect of Si increasing P uptake, which was also observed in sugarcane (Teixeira et al., 2020) and quinoa (Lata-Tenesaca et al., 2021). It can be explained by the fact that Si can regulate the expression of P transporting genes associated with the increase in the exudation rate of organic acids by the plant responsible for improving the P uptake and availability in the soil (Kostic et al., 2017).

The initial underlying benefit of Si on plant life may be involved in modifying the C:N:P stoichiometry, as there was previous evidence in a short-term study in the tillering phase in the sugarcane crop (Teixeira et al., 2020). However, there was a lack of information regarding the crop production phase. This gap was filled because in this study, in the sugarcane stem formation phase, the Si supply, by increasing the content in the plant, also modified the C:N:P stoichiometry in both water regimes. It was evidenced by the fact that there was a decrease in C and N concentrations in the leaves, except for C in PWR in Eutrophic Red Latosol, and an increase in Si concentrations. These facts contributed to the decrease of the C:P, C:Si, and N:P stoichiometric ratios, and at the same time, there was an increase in the C:N stoichiometric ratio (Figure 2). These results reinforce the Si’s ability to modify these nutrients’ homeostatic balance (Viciedo et al., 2019).

Thus, we found that the stoichiometric changes seen from the C:P, C:N, C:Si, and N:P ratios promoted by Si fertigation in the two water conditions, except for the C:P ratios in Eutrophic Red Latosol and, in AWD condition, in Quartzarenic Neosol, were sufficient to increase the C, N, and P nutritional efficiency (Figures 4, 5). It increased the ability of sugarcane plants to use nutrients in their metabolism (Prado, 2021), as stoichiometric modifications alter ecological interactions due to the role of these nutrients in the plant’s biological and biochemical functions (Prado and Silva, 2017).

The Si role in sugarcane production is an important topic verified by many authors (Camargo et al., 2017, 2019, 2021b; Marchiori et al., 2017; Bezerra et al., 2019). However, there was a lack of greater understanding of the underlying mechanisms that allow a better understanding of the stoichiometric and nutritional components in the plant’s response, not just under stress but also without stress.

Our study showed for the first time that fertigation with Si in sugarcane without stress was responsible for gains in leaf biomass, which reflected in stems in sugarcane plants cultivated in Quartzarenic Neosol and Eutrophic Red Latosol, except in Dystrophic Red Latosol (Figure 5D). This result confirms that plant responses change depending on the cultivated soil. The absence of an increase in stem dry mass in Dystrophic Red Oxisol may be more related to lower Si absorption than the other evaluated tropical soils. There was lower Si content in the stems with a 28 and 23% reduction compared to the cultivated plants Quartzarenic Neosol and Eutrophic Red Latosol, respectively (Table 3). The lack of response in this soil may be associated with a high P concentration (Table 1), with high adsorption of this nutrient occurring in highly weathered tropical soils, such as the Eutroferric Red Latosol. In the soil, Si can compete with phosphate compounds for binding sites on minerals, thus reducing the concentration of the beneficial element in the soil solution (Schaller et al., 2021) and reducing plant uptake. This fact can be observed by the P content in the plants between the two Latosols, which have greater P fixing powers, and it is not recommended to compare to Quartzarenic Neosol due to their lower capacity to fix P (low amount of clay) (Table 3). On the other hand, our study clarifies the possible mechanisms through which Si acts on sugarcane cultivated even without water deficit, indicating the establishment of a new C:N:P homeostatic balance responsible for increasing the nutrient use efficiency and providing higher dry mass biosynthesis. Overall, this is confirmed by multivariate analysis (PCA and hierarchical clustering), indicating a high similarity of dry mass production with Si fertigation in plants without stress, and an association with increased C and N use efficiency, the C, N, and P contents, and the decrease of the C:P, C:Si, and N:P stoichiometric ratios (Figures 6, 7).

According to the multivariate analysis, under water deficit, there was something similar indicating a strong relationship between the C use efficiency and the dry mass production in leaves and stems in the three tropical soils studied associated with fertigation with Si. It revealed that Si increases C use efficiency to increase dry mass biosynthesis in plants. This effect is probably due to the plant’s metabolic economy strategy since C needs 10 to 20 times more energy than Si to be incorporated into the organic skeleton. A 1% increase in Si in the biomass composition promotes a C decrease from 1.3 to 5.9% (Xia et al., 2020).

It should be noted that, for the first time, using soluble sources via fertigation with Si is also proven efficient in mitigating the damage caused by the water deficit regime in different soils (Figure 5), which is similar to studies using high Si doses incorporated into the soil (Camargo et al., 2017, 2021b; Clemente et al., 2019). However, most studies with this element do not compare the differences in plant response between soils since they study only one soil.

Our studies indicated a difference in the response of plants to the Si supply in different soils. Such effects reinforce that using the fertigation strategy with Si in a water deficit regime is more beneficial in tropical soils with low Si availability in the soil solution, such as Quartzarenic Neosol. The cluster analysis also confirmed the soil type effect on sugarcane cultivation, indicating that in Quartzarenic Neosol and Eutrophic Red Latosol, there were similar responses for Si fertigation for the variables evaluated in the stem, as it reduced the biomass losses with water deficit. Meanwhile, plants cultivated in Dystrophic Red Latosol were different. Our results show that dry mass production in the stem under water deficit conditions was associated with an increase in the Si concentration and content and a decrease in the C:Si and C:P stoichiometric ratios, especially in sugarcane plants grown in Quartzarenic Neosol and Eutrophic Red Latosol (Figure 6). It indicates that the lower Si absorption, compared to plants grown in another soil (Table 3), was not enough to change these stoichiometric ratios and express the high level of benefits. This differential Si response in sugarcane in different soils indicates the importance of edaphic characteristics in Si management in agricultural systems, such as mineralogical composition, texture, Fe and Al oxide contents, and organic matter, which are factors that can alter the success of Si fertigation and the highest stem yield (Camargo and Keeping, 2021).

Considering the results, it was clear that the first hypothesis can be accepted since Si, after fertigation application and uptake, is sufficient to modify C, N, and P stoichiometric homeostasis in leaves and stems. However, the second hypothesis tested can be partially accepted. Although Si induced stoichiometric modification, it was sufficient to increase nutritional efficiency and alleviate the losses in dry mass production of leaves and stems of sugarcane cultivated under water deficit. It also increases dry mass production of leaves and stems in the irrigated crop without water deficit, but it did not occur in all soils. In Dystrophic Red Latosol, Si benefits were limited to increasing stem production, especially in crops without water deficit. Therefore, Si use is not indicated in this soil type in properly irrigated sugarcane areas.

Our discovery enables adequate Si use, via fertigation, to increase the productivity of the sugarcane crop under water stress and without stress, depending on the cultivated soil, allowing a better understanding of the underlying Si mechanisms involved.

## Conclusion

The biological losses caused by water deficit in sugarcane plants are related to the homeostatic imbalance of C:N:P, causing reductions in nutrient use efficiencies and directly impacting dry mass biosynthesis. Silicon becomes a viable agronomic alternative in sugarcane crops with water restrictions, reducing the biological losses of water deficit, modifying the homeostatic balance of C:N:P, improving the use efficiency of these nutrients, and improving plant performance. Using Si in sugarcane cultivation without stress also proved to be efficient in improving plant performance, alternating homeostatic nutritional balance, increasing the C, N, and P use efficiencies, and increasing the dry mass production of leaves and stems.

The responses of sugarcane plants to Si application are limited depending on the type of cultivation soil, altering the intensity of responses to the benefits of the beneficial element. The strategic use of Si fertigation in a water deficit regime becomes more advantageous in tropical soils with low availability of Si in the soil solution, such as Quartzarenic Neosol. The research perspective is that Si can contribute to the sustainable cultivation of sugarcane in tropical soils in irrigated regions or under a water deficit regime.

## Data availability statement

The original contributions presented in this study are included in the article/supplementary material, further inquiries can be directed to the corresponding author.

## Author contributions

MdS and MC did the conceptualization, performed the data curation and formal analysis, investigated the data, performed the methodology, wrote the original draft of the manuscript and wrote, revised, and edited the manuscript. RdM and LP did the conceptualization, undertook the acquisition of funding, administration of the project, resources, executed the methodology, supervised the data, and wrote, revised, and edited the manuscript. JdS carried out the formal analysis, investigated the data, carried out the methodology, and wrote the original version of the manuscript. MdC carried out the acquisition of resources and financing and wrote the original version of the manuscript. All authors contributed to the article and approved the submitted version.
